# Case of Abscess in the Left Lateral Lobe of the Cerebellum, Successfully Evacuated

**Published:** 1901-06

**Authors:** J. Michell Clarke, C. A. Morton

**Affiliations:** Physician to the Bristol General Hospital; Surgeon to the Bristol General Hospital.


### CASE OF ABSCESS IN THE LEFT LATERAL LOBE OF THE CEREBELLUM, SUCCESSFULLY EVACUATED:

### WITH SOME REMARKS ON THE METHOD OF OPERATION EMPLOYED.

BY

J. MICHELL CLARKE, M.A., M.D. Cantab., F.R.C.P. Lond.,
*Physician to the Bristol General Hospital,*

AND C. A. MORTON, F.R.C.S.,
*Surgeon to the Bristol General Hospital.*

---

In view of the infrequency of recovery from cerebellar abscess, the following case is of interest; the clinical symptoms closely corresponded with those given by Dr. Acland and Mr. Ballance in their paper[1]:—

ACCOUNT OF THE CASE BY DR. MICHELL CLARKE.

The patient was a girl, æt. 13, who had never had any serious illness before the present one, and had always been bright and intelligent. Her father died of consumption, but otherwise the family was a healthy one.

The illness began with nasal catarrh, followed by a discharge from the left ear, four months previously; it was not painful, and appeared for a few days, and then passed off to reappear in a day or two. About one month previous to this she was stated to have had a "cast in her eyes," which gradually increased. The mother stated that three months previously she was brought home from school on a Thursday with pains in her forehead, mostly on the right side, and vomiting, and went to bed; on the Monday week she became unconscious,

[1] *St. Thomas's Hosp. Rep.*, 1896., xxiii. 133.

and remained so until Wednesday, when her ear "went pop," and this was followed by a large amount of discharge. The squint then disappeared, and has not been noticed since. On the Monday she began to suffer from fits. The fits were preceded by a feeling of chilliness; her face became red, she screamed out; her knees were drawn up to her chin, her arms were extended over the head and the hands clenched; all the limbs were rigid, and the body bent towards the right side. She did not bite her tongue, but passed water during the fits, and as she came round she nearly always said, "Oh, my poor head." The fits last from two to three minutes; she has had as many as seven in succession. Ever since this attack she has suffered from these fits frequently, from continuous headache, and from one or two attacks of vomiting daily, and was continually crying out with the pain in her head.

She was admitted on April 4th; her temperature was 97°, and pulse 84. On April 5th she was examined carefully; she was extremely emaciated, and very slightly deaf in the left ear, from which there was a slight discharge. She answered slowly and deliberately to questions, after pausing a while; but her answers were quite sensible and intelligent, and she spoke clearly and well. She very distinctly indicated the right frontal region as the seat of pain. She was lying on her right side, the right hand under the head, the right arm flexed, the left arm across the body, and the legs curled up on the trunk. To this position she returned when disturbed. Temperature, 97°; pulse, 84, feeble, regular; respiration, 20, easy; retention of urine and fæces; she had vomited three times since admission; she had had no fits and no rigors. She occasionally called out, "Oh, my head."

There was no squint, the eyes deviated slightly to the right; there was nystagmus when the eyes moved into either canthus, the largest excursions being to the left. The pupils were of normal size, equal, and acted well. The left palpebral fissure was a little larger than the right. Double optic neuritis, most marked on right side.

The tongue was thickly furred; she put it out straight. There was no difficulty in swallowing. The grasp of the right hand was fair, though weak; that of the left hand was very weak and feeble. There was some oscillatory tremor on movement of the left arm and hand, but she could raise it above her head.

She could not stand, and when placed on her legs fell to the right; this was tried twice. The muscles of both arms and legs were much wasted. Sensation was everywhere normal to touch and pain.

Abdominal and plantar reflexes normal (flexion of toes in latter); knee-jerks present, left perhaps slightly the greater, they were not exaggerated, and there was no clonus and no muscular rigidity.

Lying down she could move both legs, and lift either of them off the bed. Kernig's sign was not present. She resented the bed-clothes being pulled off, and wished to be covered up again, otherwise she lay passive during examination. Vomiting ceased after she was put on peptonised milk and beef-tea. The symptoms remained the same until the operation.

The general signs of intracranial abscess were thus present—namely, subnormal temperature, headache, optic neuritis, convulsions, slow cerebration, and constant vomiting.

The localising signs were: the position in bed, the marked paresis and tremor of left arm (same side), the direction of the eyes to the opposite side and nystagmoid jerkings, the weakness of both legs and tendency to fall to the right (opposite side), the headache being so distinctly on the right (opposite) side of the forehead, perhaps the greater intensity of optic neuritis on the right side, all indicating a lesion in the left lateral lobe of the cerebellum; the fact that the discharge came from the left ear was also in favour of this; and the absence of anæsthesia, of hemiplegia or of hemiplegic distribution of paresis, of paralysis of the third nerve, and of aphasia showed that the temporo-sphenoidal lobe was not involved.

A further special point in the case was the total cessation of vomiting when the patient was placed on peptonised milk and beef-tea; this in the presence of a large, probably actively extending abscess, is unusual, and might have been misleading if the other signs had not been distinct. I accordingly asked Mr. Morton to see the patient with a view to operation.

ACCOUNT OF THE OPERATION AND AFTER PROGRESS OF THE CASE, WITH REMARKS ON THE METHOD OF EXPLORATION EMPLOYED, BY MR. MORTON.

On April 6th I explored the left side of the cerebellum by Dean's method. A small flap was turned down behind the left ear, and the skull in the region of the lateral sinus removed with a $\frac{3}{4}$-inch trephine and Hoffmann's forceps. A small area of the dura mater of the cerebellar fossa just below the lateral sinus was then opened, and the cerebellum explored with a cannula and blunt trochar. I first passed the instrument backwards twice with negative result; then towards the middle lobe, also with negative result; but on pushing it forwards, inwards and downwards, I evacuated about two ounces of pus. The track of the cannula was dilated with sinus forceps, but no sloughs came away. A rubber drainage tube was left in the abscess cavity. During the administration of the chloroform before the operation was commenced it was observed that the breathing was extremely slow.

With the exception of some vomiting and headache on the 12th, she had no cerebral symptoms after the operation. The pulse the day after was 80, and on the day following 90, and R. 16, whereas the pulse the afternoon before operation was 64. There was no pyrexia after operation. The drainage tube was left out five weeks after operation, and a week later the patient was discharged from hospital. On June the 13th the sinus was quite healed. Otorrhœa on the left side was noticed at the time of the operation, and persisted until she left the hospital. On June the 13th it had ceased, and had not returned when seen later.

It will be noticed in reading the record of this case that I explored the brain by Dean's method. I always do so in these cases, for by this method both the temporo-sphenoidal lobe and the cerebellum can be readily explored from the same trephine opening. The disc of bone is removed over the lateral sinus, and then to explore the temporo-sphenoidal lobe the bone is cut away for a short distance above it, and to explore the cerebellum, below it. Thus the exploration can be carried out on both sides of the tentorium. Dean trephines $1\frac{1}{4}$ inches behind and quarter of an inch above the centre of the meatus. In order to avoid the ridge of bone which is the continuation of the superior border of the petrous portion of the temporal bone, I prefer always to trephine a little further back and higher up, $1\frac{1}{2}$ inches behind and three-quarters of an inch above the centre of the meatus. I find the presence of this ridge makes it difficult to trephine out the disc of bone at Dean's spot, as the depth of the bone is so unequal over the area trephined. I need hardly point out the great advantage of being able by the same trephine opening to explore the two common situations for brain abscess, from middle-ear disease. In many cases it is quite impossible to be sure in which the abscess lies, and failure to find pus in one situation has often prevented a further exploration of others on the ground that the patient was not in a condition for a more prolonged search by another incision and trephining, and thus the abscess has been missed.

Another method of exploration which I used in this case, and have always used in such cases, is the exploration of the brain by means of the cannula of an aspirator containing the blunt clearer supplied with it. This blunt instrument is pushed through the cerebral tissue to the depth it is intended to explore. The blunt trochar is then withdrawn, and if pus is present it will flow out, or if in withdrawing the cannula it passes through pus the latter will flow out. If a hollow needle or an empty cannula is thrust into the brain it tends to become blocked with brain tissue, and then even if it enters a collection of pus, the pus will not flow out. Macewen pointed this out in his book on *Pyogenic Diseases of the Brain.* I use a blunt trochar rather than a sharp one, as with it we could have no difficulty in penetrating brain tissue, even when surrounding an abscess cavity, and it should not penetrate any large cerebral vessel it might come in contact with. The result of the exploration in this case shows the need for repeated puncture. It was not until the fifth puncture that pus was evacuated.

